# DNA Detection of *Schistosoma japonicum*: Diagnostic Validity of a LAMP Assay for Low-Intensity Infection and Effects of Chemotherapy in Humans

**DOI:** 10.1371/journal.pntd.0003668

**Published:** 2015-04-13

**Authors:** Jing Xu, Zhi-Xun Guan, Bo Zhao, Yan-Yan Wang, Yun Cao, Hui-Qin Zhang, Xing-Quan Zhu, Yong-Kang He, Chao-Ming Xia

**Affiliations:** 1 Department of Parasitology, Medical College of Soochow University, Suzhou, Jiangsu Province, China; 2 State Key Laboratory of Veterinary Etiological Biology, Key Laboratory of Veterinary Parasitology of Gansu Province, Lanzhou Veterinary Research Institute, Chinese Academy of Agricultural Sciences, Lanzhou, Gansu Province, China; 3 Hunan Provincial Institute of Schistosomiasis Control, Yueyang, Hunan Province, China; University of Melbourne, AUSTRALIA

## Abstract

**Background:**

Schistosomiasis has decreased significantly in prevalence and intensity of infection in China, thus more accurate and sensitive methods are desperately needed for the further control of schistosomiasis. The present work aimed to assess the utility of the loop-mediated isothermal amplification (LAMP) for detection of light intensity infection or false-negative patients and patients post-treatment, targeting the highly repetitive retrotransposon SjR2 of *Schistosoma japonicum*.

**Methodology/ Principal Findings:**

LAMP was first assessed in rabbits with low intensity infection (EPG<10). Then 110 patient sera from Hunan Province, China, and 47 sera after treatment by praziquantel were used to evaluate the diagnostic validity of LAMP. Meanwhile, 42 sera from healthy individuals in a non-endemic area, and 60 sera from "healthy” residents who were identified as being negative for feces examination and immuno-methods in an endemic area were also examined. The results showed that LAMP could detect *S*. *japonicum* DNA in sera from rabbits at 3^rd^ day post-infection. Following administration of praziquantel, the *S*. *japonicum* DNA in rabbit sera became negative at 10 weeks post-treatment. Of 110 sera from patients, LAMP showed 95.5% sensitivity, and even for 41 patients with less than 10 EPG, the sensitivity of LAMP still reached to 95.1%. For 47 patients after treatment, the negative conversion rate of *S*. *japonicum* DNA in patient sera increased from 23.4%, 61.7% to 83.0% at 3 months, 6 months and 9 months post-treatment, respectively. No false-positive result was obtained for 42 human sera from non-endemic area, while for the 60 “healthy” individuals from endemic area, 10 (16.7%) individuals were positive by LAMP, which suggested that these individuals might be false-negative patients.

**Conclusions/ Significance:**

The present study demonstrated that the LAMP assay is sensitive, specific, and affordable, which would help reduce schistosomiasis transmission through targeted treatment of individuals, particularly for those with negative stool examinations who may yet remain infected. The LAMP assay may provide a potential tool to support schistosomiasis control and elimination strategies.

## Author Summary

Accurate diagnostic tests play a key role in patient management and control of schistosomiasis, especially in China where the prevalence and intensity of *Schistosoma japonicum* infection is low in recent years. The present study aimed to assess the utility of the loop-mediated isothermal amplification (LAMP) assay for detection of light intensity infection or false-negative patients and for the evaluation of chemotherapy effectiveness in patients, targeting the highly repetitive retrotransposon SjR2 of *S*. *japonicum*, using 110 serum samples of schistosomiasis patients. The results showed that the LAMP assay had high sensitivity of 95.1% for the diagnosis of *S*. *japonicum* infection with the lowest intensity (EPG<10). For the assessment of efficacy after treatment with praziquantel, the negative conversion rate increased from 23.4%, 61.7% to 83.0% at 3 months, 6 months and 9 months post-treatment by LAMP, whereas for ELISA and IHA, the negative conversion rate remains at a low level (25.5% by ELISA and 31.9% by IHA) even at 9 months after treatment. Our results demonstrated that the LAMP assay may provide a valuable tool for the diagnosis of *Schistosoma* infection, especially for cases of light infection which is coincident with the current endemic situation.

## Introduction

Schistosomiasis remains one of the most important chronic parasitic diseases in tropical regions and affects approximately 200 million people, despite the continued implementation of control measures [1]. Schistosomiasis japonica, caused by infection with *Schistosoma japonicum*, is mainly endemic in China. In areas where infection occurs, the prevalence and infection intensity is now low due to long-term and large-scale chemotherapy campaigns [2]. Methods that allow infections to be correctly diagnosed are a prerequisite for effective disease control. All present schistosomiasis control measures, including targeted treatment of all infected individuals, especially those with low-intensity infections, and large-scale surveillance of disease transmission are strongly dependent on sensitive and accurate diagnostic assays [3]. Indeed, the move to identify and evaluate highly sensitive diagnostics has become increasingly necessary as the prevalence and intensity of schistosome infections continues to decline worldwide [4]. The Kato-Katz fecal smear technique is the most commonly used method to diagnose schistosomiasis. However, various studies have shown that its sensitivity is less appropriate for low endemic areas, post-treatment situations, and for determination of incidence [5,6,7]. Moreover, traditional stool examinations always underestimate the prevalence of schistosomiasis due to their low diagnostic sensitivity [8]. Antibody detection methods generally have high sensitivities, but the slow reduction of specific antibody levels after treatment and the inability to discriminate between active and past *S*. *japonicum* infection constituted great disadvantages of the antibody-based assays [9,10]. Moreover, the level of antibodies persisted after treatment, which could not indicate whether the patients were cured or not [11,12]. In recent years, detection of circulating antigens, such as circulating anodic antigens (CAA) in serum or urine, seems a promising tool for diagnosis of *Schistosoma* infection [7,13]. A field study done by van Dam et al. [7], using lateral-flow assay for determination of CAA in urine and serum, showed that the method was at least 6 times more sensitive than triplicate Kato-Katz thick smears. However, due to the lack of “golden” standard reference test, different diagnostic methods are not comparable, thus a more sensitive standard is still needed [14].

With the development of molecular techniques, polymerase chain reaction (PCR)-based methods have shown great sensitivity and specificity for detection of *Schistosoma* DNA in a variety of samples [9,10,15–23]. However, the dependence on expensive apparatus and on specialized training in molecular biology restricts their widespread applications for field conditions. As an alternative, the loop-mediated isothermal amplification (LAMP) may provide a potential tool for diagnosis of schistosomiasis. The LAMP assay, firstly reported by Notomi et al. [24], has been rapidly accepted for detection of various pathogens, including *Plasmodium falciparum*, *S*. *mansoni*, *S*. *haematobium* and *S*. *japonicum*, due to its simplicity and rapidity [25–32]. This technique does not require sophisticated equipment for DNA amplification or for amplicon detection [33], which is of great value for field use. Our previous study established a LAMP assay based on the sequence of highly repetitive retrotransposon SjR2, which is able to detect 0.08 fg *S*. *japonicum* DNA, and 10^4^ times more sensitive than conventional PCR [30].

To extend from our previous work, in this study, we assessed the utility of this LAMP assay for detection of light infection or false-negative patients of schistosomiasis and evaluation of chemotherapy efficacy, with the aim of providing a potential tool to support schistosomiasis control and elimination strategies.

## Materials and Methods

### Ethics statement

This study was funded by A Project Funded by the Priority Academic Program Development of Jiangsu Higher Education Institution(No.YX13400214); the National Basic Research Program of China(973 Program, Grant No. 2007CB513100); Key Laboratory of Control and Prevention of Parasitic Disease of Healthy Ministry, No. wk014-001). The funders had no role in study design, data collection and analysis, decision to publish, or preparation of the manuscript.

### Establishment of light-infection rabbit models and sample collections

This study was carried out in strict accordance with the recommendations in the Guide for the Care and Use of Laboratory Animals of the National Institutes of Health. The protocol was approved by the Committee on the Ethics of Animal Experiments of the Soochow University (Permit Number: 2007–13). All surgery was performed under sodium pentobarbital anesthesia, and all efforts were made to minimize suffering.

*Schistosoma japonicum*-infected snails (*Oncomelania hupensis*) were obtained from Jiangsu Institute of Parasitic Diseases, China. The living snails were putting into a breaker filled with 4/5 volume of water and then exposed to a light source to induce shedding of live *S*. *japonicum* cercariae.

**Fig 1:**
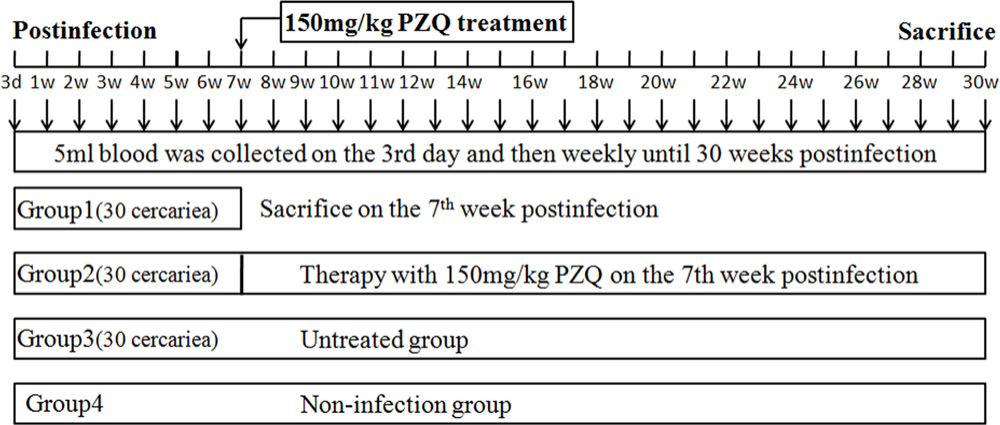
Schematic representation of rabbit infections and blood sample collections.

Eight adult female New Zealand rabbits, weighing 2.0–2.5 kg, were randomly divided into four groups of two rabbits each. Each rabbit in Group 1 was percutaneously infected with 30 mixed sexual cercariae. On the 7th week post-infection, all the rabbits were sacrificed, and adult worms (male and female) were collected to confirm the real intensity of infection. Rabbits of group 2 and group 3 were given the same infective intensity as group1. Rabbits of group 4 administrated with saline were used as uninfected control. The faecal samples of rabbits for Kato-Katz egg examination were collected weekly from 1 week post-infection to 30 weeks post-infection. Rabbits in group 2 were treated with a single dose of 150 mg praziquantel per kilogram body weight on the 7th week post-infection. This dose was known to eliminate *S*. *japonicum* in our model [9,30]. Rabbits of group 3 remained as untreated control, and received no drug treatment. Blood samples from rabbits of group 2 to group 4 were collected on the 3rd day and then weekly until 30 weeks post-infection (Fig 1). Serum of each blood samples was separated by centrifugation (2,000 rpm for 10 min) after storage at 37°C for 2 h. All of the serum samples were stored at -20°C until use. On the 30th week post-infection, all of the rabbits were sacrificed, and the portal system and liver of the rabbits in group 2 were examined for evaluation of therapy efficacy.

### Collection of field serum samples

One hundred and ten serum samples positive for *S*. *japonicum* infection determined by triplicate Kato-Katz stool examinations were obtained from 30 females and 80 males living in endemic areas in Hunan Province, China. Written consent letters were obtained from all participants. Ethical approval was obtained from the village (local government), county (anti-schistosomiasis office) and provincial (schistosomiasis headquarters) authorities. Five mL blood sample was collected from each participant, and about 2 mL serum was obtained from each blood sample. Then the serum samples were divided into 3 groups according to the eggs per gram of feces (EPG): 93 samples with <100 EPG, 13 samples with 100–400 EPG, 4 samples with >400 EPG. According to the new classification in China, which sets 100 EPG as the lower limit for a heavy infection; 40–99 EPG denotes a moderate infection, while 40 EPG is the upper limit for a light infection [34], the 93 samples with less than 100 EPG was divided again: 10 samples with 40–99 EPG, 42 samples with 10–39 EPG, 41 samples with < 10 EPG (Table 1). All the 110 cases were treated with praziquantel (single oral dose of 40 mg/kg), two stool samples were obtained from each participant at the 3rd month, the 6th month and the 9th month post-treatment, respectively, and each sample was subjected to triplicate Kato-Katz thick smears to evaluate the effectiveness of chemotherapy. Meanwhile, 2 mL serum samples were collected from the study population accordingly. However, only 47 serum samples from the participants had the complete data records. Forty-two serum samples from uninfected individuals collected from Wuxi, Jiangsu Province, China were used as negative control to evaluate the specificity of the LAMP assay. Meanwhile, 60 serum samples from individuals living in fishing villages in endemic areas, which were negative for fecal eggs by triplicate Kato-Katz thick smears, and also negative for serology diagnosis by triplicate IHA and ELISA tests, were used to evaluate the validity of the LAMP assay.

**Table 1:** General characteristics of the study population.

EPG	No. male (mean age and range)	No. female (mean age and range)	Total population (mean age and range)
>400	4 (21.5,11–53)	0	4 (21.5,11–53)
100–400	11 (51.8,27–70)	2 (35.0,23–47)	13 (49.2,23–70)
<100	65 (46.3,9–74)	28 56.0,39–70)	93 (49.5,11–53)

### DNA extraction

DNA from all of the collected serum samples was extracted using the method described previously [9]. Briefly, 200 μl sera were dissolved in 400 μl serum lysis buffer, incubated 20 min at 55°C, and then extracted with phenol–chloroform–isoamyl alcohol (25:24:1) and precipitated with dehydrated alcohol.

### IgG-enzyme linked immunosorbent assay (IgG-ELISA)

Serum samples were tested for anti-*Schistosoma* antibodies by IgG-ELISA. Serology was performed according to the protocol described by Xia et al. [9]. Sera were considered positive when the OD value exceeded the mean±3 SD absorbance of sera from non-infected samples.

### Indirect hemagglutination assay (IHA)

Indirect hemagglutination kit containing human erythrocytes coated with soluble egg antigen is commercially available from the Anhui Provincial Institute of Parasitic Diseases (Wuhu, China). The test procedure followed a previous study [2]. The test result was considered positive when a positive reaction appeared at a titer 1:10.

### LAMP

The LAMP assay was based on a previously study targeting the highly repetitive retrotransposon SjR2 of *S*. *japonicum* (GeneBank Accession no. AF412221) [9,30,35], with slight modifications. The LAMP assay was carried out with a total of 25 μl reaction mixture containing 2.5 μl 10×Bst-DNA polymerase buffer, 6 mmol/L MgSO4, 1.4 mmol/L dNTP, 0.2mmol/L F3, B3, 1.6 mmol/L FIP, BIP, 0.8 mol/L betain, 8 U Bst-DNA polymerase, and 5 μl template DNA. The reaction mixture was incubated at 64°C for 90 min. The LAMP amplification results were identified by adding 5 μl 1:80 diluted 10000×SYBR Green I after incubation, positive reactions were detected by an orange to green color change visible under normal light.

### Data management and statistical analysis

Diagnostic sensitivity, specificity, PPV and NPV were calculated using the following formulae:

Sensitivity = True positives/(True positives+False negatives)
Specificity = True negatives/(True negatives+False positives)
Positive predictive value (PPV) = True positives/(True positives+False positives)
Negative predictive value (NPV) = True negatives/(True negatives+False negatives)

The SPSS 19.0 software was used for data analysis.

## Results

### Confirmation of real intensities of infection and therapy efficacy in rabbit models

On average, 3 female and 10 male adult worms (3 pairs of worms) were collected in rabbits of Group 1, and the mean EPG of the rabbits infected with 30 cercariae was 16, which is very low intensity infection. In addition, for fecal examinations of the rabbits, the eggs could not be found untill 7 weeks post-infection from group 1 to group 3. No adult worms were found in the portal system and liver of the rabbits in group 2, indicating thoroughly treatment of the rabbits in group 2. While for the rabbits without praziquantel treatment, 3 female and 18 male adult worms were found, and egg-granulomas were clearly observed (Table 2).

**Table 2:** Detection results of EPG, LAMP, IHA, ELISA and worm burden in rabbit experiments after infection of *Schistosoma japonicum* cercaiea.

Post-infection	Group 1*				Group 2				Group 3			
	No.EPG	(positive rate, %)			No.EPG	(positive rate, %)			No.EPG	(positive rate, %)		
		LAMP	IHA	ELISA		LAMP	IHA	ELISA		LAMP	IHA	ELISA
3d	None**	50	0	0	None	50	0	0	None	50	0	0
1w	None	50	0	0	None	50	0	0	None	50	0	0
2w	None	100	0	0	None	100	0	0	None	50	0	0
3w	None	100	0	0	None	100	0	0	None	50	0	0
4w	None	100	0	0	None	100	0	0	None	100	0	0
5w	None	100	100	0	None	100	100	0	None	100	0	0
6w	None	100	100	100	None	100	100	100	None	100	100	100
7w	16	100	100	100	8	100	100	100	16	100	100	100
8w					8	100	100	100	16	100	100	100
9w					None	100	100	100	8	100	100	100
10w					None	100	100	100	None	100	100	100
11w					None	100	100	100	8	100	100	100
12w					None	100	100	100	8	100	100	100
13w					None	100	100	100	None	100	100	100
14w					None	100	100	100	None	50	100	100
15w					None	50	100	100	None	100	100	100
16w					None	50	100	100	None	100	100	100
17w					None	0	100	100	None	100	100	100
18w					None	0	100	100	None	100	100	100
19w					None	0	100	100	None	50	100	100
20w					None	0	100	100	None	50	100	100
21w					None	0	100	100	None	100	100	100
22w					None	0	100	100	None	100	100	100
23w					None	0	100	100	None	100	100	100
24w					None	0	100	100	None	100	100	100
25w					None	0	100	100	None	100	100	100
26w					None	0	100	100	None	50	100	100
27w					None	0	100	100	None	100	100	100
28w					None	0	100	100	None	100	100	100
29w					None	0	100	100	None	50	100	100
30w					None	0	100	100	None	100	100	100
Male worms	10				0				18			
Female worms	3				0				3			

*All of the rabbits in Group 1 were sacrificed on the 7th week post-infection.

**None means no egg was found by triplicate Kato-Katz thick smears in three fecal samples.

### Serum DNA detection in rabbit models with light infection of *S*. *japonicum*

**Fig 2:**
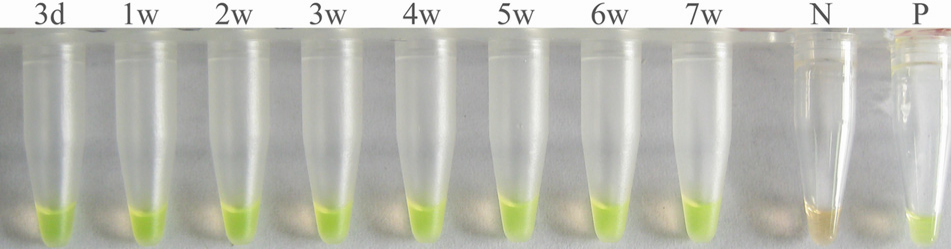
Detection of *Schistosoma japonicum* DNA in sera of rabbits with light-infection by LAMP. Serum samples were collected at 3 days (3d) and 1–7 weeks (1w–7w) post-infection. Tube N, serum of non-infected rabbits; tube P, DNA of *S*. *japonicum* adult worm (positive control).

**Fig 3:**
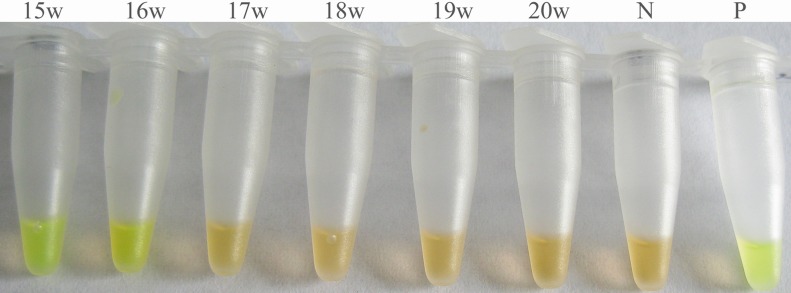
Detection of *Schistosoma japonicum* DNA in sera of rabbits after treatment with praziquantel by LAMP. Serum samples were collected from 15weeks to 20 weeks post-infection (8–13 weeks post-treatment). Tube N, serum of non-infected rabbits (negative control); tube P, DNA of *S*. *japonicum* adult worm (positive control).

ELISA and IHA examination of rabbit serum samples gave positive results at 5 weeks and 4 weeks post-infection, respectively, and the antibody sustained at high level even at 23 weeks post-treatment (30 weeks post-infection, Table 2). Whereas the *S*. *japonicum* DNA was detectable by LAMP at the 3rd day post-infection in serum of rabbit model with very low intensity infection (EPG = 16, Table 2, Fig 2). Following administration of praziquantel, the detection of *S*. *japonicum* DNA in rabbit sera became negative at 10 weeks post-treatment (17 weeks post-infection, Table 2, Fig 3).

### Validity of LAMP for diagnosis of schistosomiasis in humans

Of the 110 patient serum samples with confirmed *S*. *japonicum* infection by stool examination, the sensitivity and specificity of LAMP assay was 95.5% and 100%, respectively. Whereas, the sensitivity and specificity of ELISA and IHA was 84.6% and 85.7%, and 91.8% and 88.1%, respectively. There was significant difference between the sensitivity of LAMP and ELISA (χ^2^ = 7.273, *P* = 0.007). Table 3 shows the PPV and NPV of the three detection methods, and the LAMP assay had the highest PPV (100%) and NPV (89.4%). Although all the three methods showed 100% sensitivity for patients with high-intensity infections (EPG>400), LAMP assay had higher sensitivity (95.7%) than ELISA (83.9%) and IHA (93.4%) for patients with <100 EPG(χ^2^ = 7.627, *P* = 0.022, Table 4). For 42 patients with 10–39 EPG, the detection rate of LAMP achieved 97.6%, which was significantly higher than the two immunoassays(χ^2^ = 6.157, *P* = 0.046, Table 5). Even for 41 patients excreting <10 EPG, which was very low-intensity infection, the sensitivity of LAMP assay sustained at a high level (95.1%), and only 2 cases were missed (Table 5).

**Table 3:** Results of ELISA, IHA and LAMP for diagnosis of 110 *Schsitosoma japonicum* positive cases by Kato-Katz method.

Methods	No. examined cases	Sensitivity (%) (95%CI)	Specificity (%) (95%CI)	PPV(95%CI)	NPV(95%CI)
ELISA	110	84.6 (77.9–91.3)	85.7 (79.2–92.2)	93.9 (89.2–98.6)	67.9 (55.3–80.5)
IHA	110	91.8 (86.7–96.9)	88.1 (82.0–94.2)	95.3 (91.3–99.3)	80.4 (68.9–91.9)
LAMP*	110	95.5 (91.6–99.4)	100	100	89.4 (80.6–98.2)

*The sensitivity of LAMP assay and ELISA showed significant difference (χ^2^ = 7.273, *P* = 0.007), and there was little difference between LAMP and IHA (χ^2^ = 1.221, *P* = 0.269), and between ELISA and IHA (χ^2^ = 2.791 *P* = 0.095).

**Table 4:** Comparison of ELISA, IHA and LAMP for diagnosis of different infection intensities of *Schsitosoma japonicum* in 110 samples.

		No. positive (sensitivity, %, 95%CI)				
Intensity of infection (EPG)	No. examined cases	ELISA	IHA	LAMP	χ^2^	***P*** value
<100	93	78 (83.9) (76.4–91.4)	85 (93.4) (88.4–98.4)	89 (95.7) (91.6–99.8)	7.627	0.022
100–400	13	11 (84.6)	12 (92.3)	12 (92.3)	/	/
>400	4	4 (100)	4 (100)	4 (100)	/	/

**Table 5:** Comparison of ELISA, IHA and LAMP for diagnosis of 93 serum samples with various low-intensity infection of *Schistosoma japonicum*.

Intensity of infection (EPG)	No. examined cases	No. positive (positive rate, %, 95%CI)				
		ELISA	IHA	LAMP	χ^2^	***P*** value
<10	41	34 (82.9, 71.4–91.4)	39 (95.1, 88.5–100)	39 (95.1, 88.5–100)	4.657	0.097
10–39	42	35 (83.3, 72.0–94.6)	36 (85.7, 75.1–96.3)	41 (97.6, 93.0–100)	6.157	0.046
40–99	10	9 (90.0)	9 (90.0)	9 (90.0)	/	/

After treatment with praziquantel, all the stool samples were negative for faecal eggs by triplicate Kato-Katz method. However, the negative conversion rate of *S*. *japonicum* specific SjR2 DNA detected by LAMP assay increased remarkably from 23.4%, 61.7% to 83.0% at 3 months, 6 months and 9 months post-treatment, while for the two immunoassays, the negative conversion rate of antibodies sustained at a low level even after 9 months post-treatment (Table 6). Statistical analysis showed that there was significant difference between LAMP and the two immunodiagnostics for the negative conversion rate of *S*. *japonica* at 6 months and 9 months post-treatment (χ^2^ = 17.63 and 37.43, both *P* value <0.0001).

**Table 6:** Comparison of negative conversion rate of ELISA, IHA and LAMP assay in 47 cases infected with *Schistosoma japonicum* after treatment with praziquantel.

Periods after treatment	No. examined cases	No. (negative conversion rate, %, 95%CI)			χ^2^	***P*** value
		ELISA	IHA	LAMP		
3 months	47	8(17.0, 6.3–27.7)	11(23.4, 11.3–35.5)	11(23.4, 11.3–35.5)	0.76	0.683
6 months	47	9(19.1, 7.9–30.3)	20(42.5, 28.4–56.6)	29(61.7, 47.8–75.6)	17.63	<0.0001
9 months	47	12(25.5, 13.0–38.0)	15(31.9, 18.6–45.2)	39(83.0, 72.3–92.7)	37.43	<0.0001

Additionally, 42 serum samples of individuals from non-endemic areas, and 60 serum samples of residents in endemic areas who were identified with negative results for all the three commonly used diagnostic methods, including triplicate stool examinations, IHA and ELISA tests, were used to assess the validity of LAMP assay for field diagnosis of schistosomiasis. No positive result was observed for 42 uninfected human serum samples, indicating an excellent specificity of LAMP assay (Table 7). However, for the 60 serum samples from residents in endemic areas, which were negative for faecal eggs and serological methods, 10 (16.7%) samples were positive diagnosed by LAMP assay (Table 7), which might be missed by the commonly used diagnostic methods, such as Kato-Katz method, IHA and ELISA.

**Table 7:** LAMP for detection of “healthy” human serum samples from endemic and non-endemic areas.

serum samples	No. examined cases	No. positive	Positive rate (%) (95%CI)
Non-endemic area	42	0	0
Endemic area	60	10	16.7 (7.3–26.1)

## Discussion

Diagnosis is central to control of schistosomiasis [36]. The prevention and control of the disease need rapid and reliable diagnostic techniques to identify target population accurately for treatment [37]. However, the currently available diagnostic assays are not ideal, since the search for eggs in stools and detection of circulating antigens lack sensitivity in low prevalence and post-treatment situations, and antibody detection lacks specificity [38], and cannot distinguish current and cured infections, which results in the difficulties in determining prevalence, identifying true infected individuals for selective chemotherapy and assessing the effectiveness of intervention including follow-up of chemotherapy [34,39,40,]. Our results further demonstrated the limitations of the direct parasitological technique (Kato-Katz method) and antibody detection assays (ELISA and IHA). In rabbit models infected with *S*. *japonicum* cercariea, we did not find faecal eggs until 7 weeks post-infection, and for the two immunoassays, the earliest positive detection result was obtained at 4 weeks post-infection. Our rabbit experiment results confirmed that both the stool examination and antibody detection methods could not give early diagnosis at the beginning of *S*. *japonicum* infection. Moreover, 4 egg-positive cases were negative for antibodies by ELISA and IHA, with ages ranging from 43 to 65, indicating that antibody detections were insufficient for diagnosis of schistosomiasis in patients of older ages, due to immune down-regulation in chronic infection stages [7,41]. However, DNA amplification assays, which had identical diagnostic value with that of parasitological methods, may provide alternative approaches for sensitive and specific diagnosis of schistosomiasis. Lier et al. [21] reported a real-time PCR assay for detection of low intensity *S*. *japonicum* infections in a pig model. Subsequently, this method was used in a clinical trial. This real-time PCR assay detected slightly more positive faecal samples than the microscopy method, but was consistently negative in serum and urine samples [22]. To our knowledge, most of the PCR-based methods were focused on the detection of specific *Schistosoma* DNA in faeces, all of these methods could be seen as improvements on the stool examination approach [7], thus the sensitivity was influenced by the large day-to-day egg fluctuations in infected individuals [42]. In general, serum and urine samples are easier to obtain and more accepted in many populations than faecal samples, and unlike eggs in faecal samples, schistosome DNA would be equally distributed throughout the serum of the patient, resolving the restrictions of uneven distributions of eggs in stool samples [43]. Therefore, the detection of *Schistosoma* DNA from serum samples would be more accurate in field conditions. In our previous study, we found that the *S*. *japonicum* DNA in host serum primarily comes from the residual body of dead schistosomula in the first 4 weeks post-infection, while during the spawning stage of the female schistosome, the parasite DNA mainly comes from the disintegration of inactive eggs [44]. Furthermore, a 230-bp sequence from the highly repetitive retrotransposon SjR2 was identified in our previous study, and showed high sensitivity and specificity for detecting *S*. *japonicum* DNA in sera of rabbit model and patients [9]. Although PCR-based methods have the potential for sensitive and specific detection of schistosomiasis, the dependence on expensive apparatus restricts their wide application in the field.

Unlike PCR, LAMP assay does not require amplification cycles by thermocycling or amplicon detection by electrophoresis. Given these features, LAMP is potentially useful for work in the field and has already used in rural laboratories in developing areas for the diagnosis of tuberculosis [45]. Our previous study established a LAMP assay targeting *S*. *japonicum* SjR2, and the method was capable of detecting as little as 0.08 fg *S*. *japonicum* DNA, which was 10^4^ times more sensitive than common PCR assay. In particular, the LAMP assay was able to detect *S*. *japonicum* DNA in rabbit sera at 1 week post-infection, and become negative at 12 weeks post-treatment [30]. However, the rabbit models used in our previous study were of high and moderate intensity infections, and the detection of *S*. *japonicum* DNA in low-intensity infections is more consistent with the current epidemiological situation.

In this study, the utility of LAMP assay was firstly assessed in rabbit models with very low grade intensities of infection (EPG = 16). It was able to detect *S*. *japonicum* DNA in serum at 3 days post-infection, and the detection results became negative at 10 weeks post-treatment, indicating that the LAMP method was useful for diagnosis of schistosomiasis, especially with low-intensity infection, and had potential for evaluation of chemotherapy effectiveness.

Then the field diagnostic value of the LAMP method, and its ability for evaluation of effectiveness of drug treatments was tested using 110 patient serum samples with confirmed *S*. *japonicum* infection by stool examination. Meanwhile, two of the most extensively used immunoassays (ELISA and IHA) in the field were also used to assess the validity of the LAMP assay for diagnosis of schistosomiasis. Our detection results showed that the LAMP assay performed better than the commonly used immunoassays in terms of higher sensitivity in patients with low-intensity infection (Table 4 and Table 5). After treatment with praziquantel, the negative seroconversion rate of IHA and ELISA sustained at low levels, while for the LAMP assay, the negative conversion rate of *S*. *japonicum* DNA in serum increased from 23.4%, 61.7% to 83.0% after 3 months, 6 months and 9months post-treatment (Table 6). All of the results confirmed that the LAMP assay was efficient for diagnosis of cases with low-intensity infections, and had potential for assessment of effectiveness of drug treatment.

Finally, 60 residents living in endemic areas with negative detection results of Kato-Katz, IHA and ELISA, were recognized as “healthy” residents, and were employed to assess the ability of LAMP assay for accurate diagnosis of schistosomiasis. Of the 60 serum samples from “healthy” individuals, 10 (16.7%) were diagnosed as positive by LAMP assay (Table 7), who might had been missed by parasitological methods, indicating that traditionally used methods lack sensitivity for diagnosis of individuals with low intensity infections. A field study done by Xu et al. [3] further confirmed our results. In this study, of 1371 enrolled residents, parasitological detection identified only 74 (5%) individuals as being egg-positive by Kato-Katz thick smears, of whom all the individuals were also positively diagnosed by LAMP detection of SjR2 DNA. More importantly, additional 368 (27%) individuals were positive for SjR2 DNA [3]. Professor Clive Shiff of Johns Hopkins Bloomberg School of Public Health, USA, commented on this paper as “New diagnostics reform infectious parasite epidemiology” [12]. The comment suggested that continuous surveillance to predict any resurgence of infection by accurate and sensitive measuring methods is highly recommended. It is very important to help reduce schistosomiasis transmission through targeted treatment of individuals, particularly those people who are presumed to be free of infection (false-negative) may actually remain infected and capable of infecting snails when their faeces get into the water.

In conclusion, the LAMP assay with rapidity, simplicity, sensitivity and specificity is suitable not only for case detections, but also for disease surveillance in schistosomiasis-endemic areas. Application of this method may improve the identification of cases with low-intensity infections and targeted treatment, which is of great significance for schistosomiasis control and elimination programmes.

## Supporting Information

S1 Checklist STARD checklist. (DOC)

S1 Flowchart STARD flowchart. (PDF)
