# Supplementary figures and images for: DNA Detection of Schistosoma japonicum: Diagnostic Validity of a LAMP Assay for Low-Intensity Infection and Effects of Chemotherapy in Humans
Source: PLoS Negl Trop Dis. 2015 Apr 13;9(4):e0003668. doi: 10.1371/journal.pntd.0003668 (PMC4395225; doi:10.1371/journal.pntd.0003668)

General example

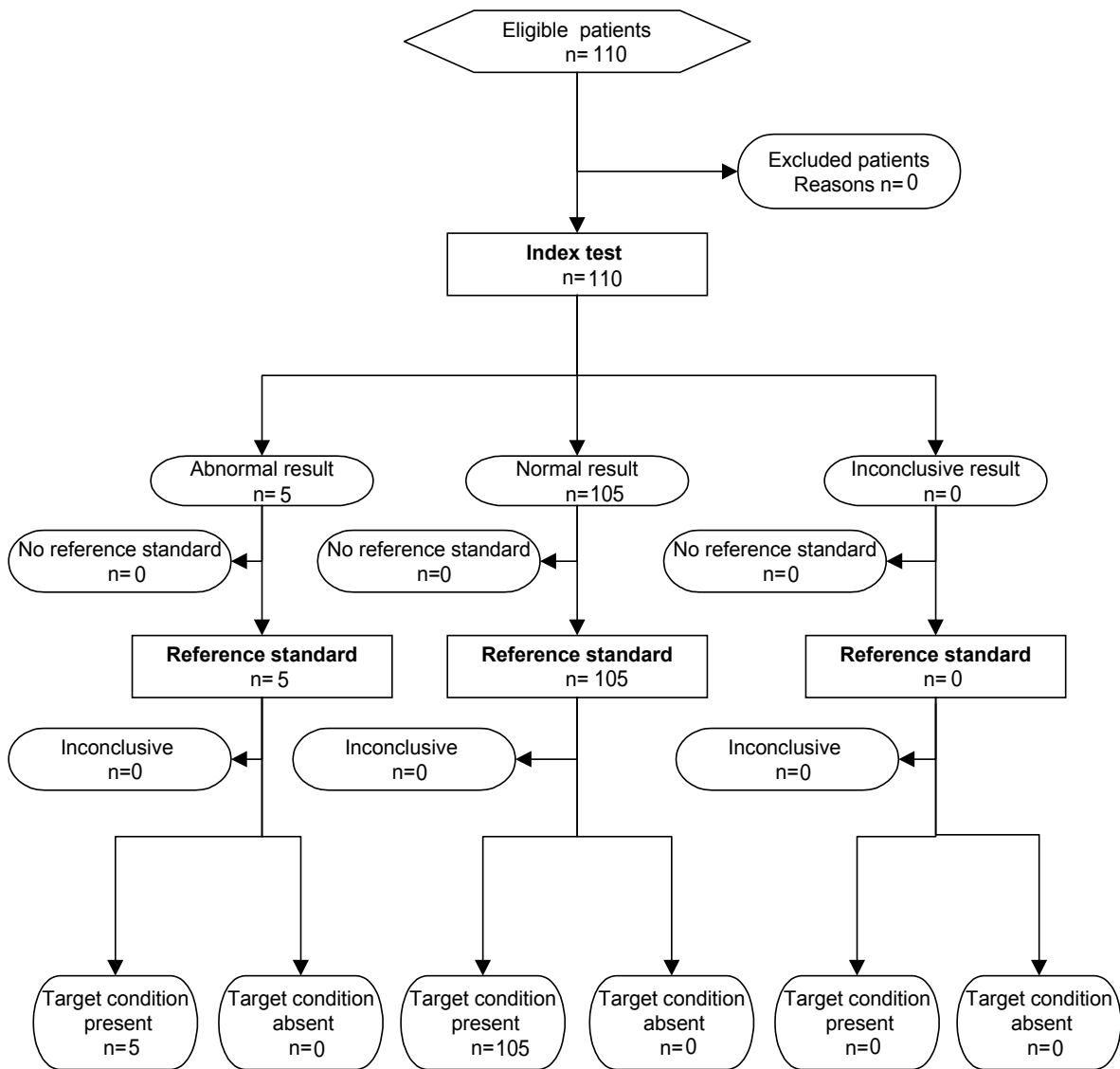

Supplement: S1 Flowchart — (PDF) [file pntd.0003668.s002.pdf]
